# Characterization of Natural and Simulated Herbivory on Wild Soybean (*Glycine soja* Seib. et Zucc.) for Use in Ecological Risk Assessment of Insect Protected Soybean

**DOI:** 10.1371/journal.pone.0151237

**Published:** 2016-03-10

**Authors:** Hidetoshi Goto, Hiroshi Shimada, Michael J. Horak, Aqeel Ahmad, Baltazar M. Baltazar, Tim Perez, Marc A. McPherson, Duška Stojšin, Ayako Shimono, Ryo Ohsawa

**Affiliations:** 1 Monsanto Japan Limited, 2-5-18, Kyobashi, Chuo-ku, Tokyo, 104–0031, Japan; 2 Monsanto Japan Limited, Kawachi Research Farm, 4475–2, Manaita Aza Dotemukai, Kawachi-machi, Inashiki-gun, Ibaraki, 300–1331, Japan; 3 Monsanto Company, 800 N Lindbergh Blvd., St. Louis, MO, 63167, United States of America; 4 Faculty of Science, Toho University, 2-2-1 Miyata, Funabashi, Chiba, 274–8510, Japan; 5 Laboratory of Plant Breeding, Graduate School of Life and Environmental Sciences, University of Tsukuba, 1-1-1 Tennoudai, Tsukuba, Ibaraki, 305–8572, Japan; Institute of Genetics and Developmental Biology, Chinese Academy of Sciences, CHINA

## Abstract

Insect-protected soybean (*Glycine max* (L.) Merr.) was developed to protect against foliage feeding by certain Lepidopteran insects. The assessment of potential consequences of transgene introgression from soybean to wild soybean (*Glycine soja* Seib. et Zucc.) is required as one aspect of the environmental risk assessment (ERA) in Japan. A potential hazard of insect-protected soybean may be hypothesized as transfer of a trait by gene flow to wild soybean and subsequent reduction in foliage feeding by Lepidopteran insects that result in increased weediness of wild soybean in Japan. To assess this potential hazard two studies were conducted. A three-year survey of wild soybean populations in Japan was conducted to establish basic information on foliage damage caused by different herbivores. When assessed across all populations and years within each prefecture, the total foliage from different herbivores was ≤ 30%, with the lowest levels of defoliation (< 2%) caused by Lepidopteran insects. A separate experiment using five levels of simulated defoliation (0%, 10%, 25%, 50% and 100%) was conducted to assess the impact on pod and seed production and time to maturity of wild soybean. The results indicated that there was no decrease in wild soybean plants pod or seed number or time to maturity at defoliation rates up to 50%. The results from these experiments indicate that wild soybean is not limited by lepidopteran feeding and has an ability to compensate for defoliation levels observed in nature. Therefore, the potential hazard to wild soybean from the importation of insect-protected soybean for food and feed into Japan is negligible.

## Introduction

Three million metric tons of soybean (*Glycine max* (L.) Merr.) are consumed in Japan annually and over 90% of the soybean grain is imported [1, 2]. In addition, genetically modified (GM) soybean accounts for more than 80% of the soybean grown in the world [3]. From these metrics, we estimated that about 70% of the soybean consumed in Japan is genetically modified.

The Ministry of the Agriculture, Forestry and Fisheries (MAFF) and the Ministry of the Environment (MOE) in Japan require extensive data for environmental risk assessment (ERA) of each GM crop prior to approval for importation for food and feed [4]. Because soybean has a cross-compatible wild relative, wild soybean (*Glycine soja* Seib. et Zucc.) in Japan, the ERA needs to include the potential consequence of transgene introgression from soybean to wild soybea.

A total of 64 different GM events across several different crops have been evaluated for environment risk and approved in Japan [4].

Risk assessment is a process where the potential for a hazard to occur is considered in the context of the likelihood of that hazard being realized. As such, risk has two distinct elements: hazard (harm or consequence) and exposure (the likelihood of the hazard to occur), each of which is characterized separately prior to the formulation of risk estimation [5]. If no meaningful hazard or exposure are identified, the risk can be estimated as “negligible.” If both hazard and exposure are identified, the risk is estimated by considering hazard in the context of exposure [5, 6].

Before undertaking an ERA for a genetically modified crop, it is critical to identify potential hazards based on knowledge of the crop, the trait being introduced, the receiving environment, and their interactions [7–9]. For the identification and evaluation of environmental risks of a genetically modified plant, the risk assessment strategy begins with established differences between the genetically modified plant relative to its conventional counterpart [5, 10]. This approach allows the risk assessors to focus on relevant differences, such as those that have a potential to affect important ecological processes, alter the weediness of the plant, or impact non-target organisms. Comparative assessment data allows the risk assessment to focus on where the receiving environment could be meaningfully impacted [5, 11, 12].

Environmental risk from the importation of GM crops into Japan is comprehensively assessed based on potential effects from: 1) competition with wild plant species, 2) toxic substance produced by GM crop, and 3) cross-breeding with related species [4]. Crops that do not have cross-compatible wild relatives in Japan, such as corn and cotton, do not require an assessment of potential effects from cross-breeding with related species as there is no potential exposure [4].

The GM soybean varieties are assessed for potential effects from cross-breeding with wild soybean, a cross-compatible relative that grows naturally in Japan [13]. Wild soybean is known to grow in Asia from 24° to 53° north latitude, and from 97° to 143° east longitude, and can be found in nature in China, the far east Russia, South Korea, and lowland regions of Japan [14]. In Japan, wild soybean grows in Hokkaido, the Main Island, Shikoku, and Kyushu, in ruderal and disturbed habitats including river beds, abandoned industrial sites, in and around cultivated fields, and along roadsides where soil is disturbed by human activities [15–18]. Wild soybean is found mainly near river basins or downriver of paddy fields in the Japanese islands of Hokkaido, Tohoku, Kinki, Chugoku, and Shikoku [19–24]. Furthermore, wild soybean populations were observed in areas disturbed by floods or human activities such as agriculture and construction [25, 26].

There are many barriers to natural hybridization between soybean and wild soybean, including the high self-pollination rates of both species [13], limited distance of pollen-mediated gene flow between wild soybean and cultivated soybean, asynchrony of flowering, and presence of pollinators. However, studies have shown that soybean and wild soybean can cross at low frequency when plants are in relative close proximity (one to a few meters) and when their flowering time overlapped [27, 28]. Thus, an ERA for GM soybean includes an assessment of the likelihood for gene flow from cultivated soybean to wild soybean to occur and the potential hazard or consequences to the environment if introgression of a transgene were to occur.

In total, 10 herbicide-tolerant soybean events have had an ERA completed and have been approved for import in Japan [4]. Each of these environmental assessments concluded that introgression of an herbicide-tolerant traits into wild soybean would not affect their populations because herbicides are not often applied (limited selection pressure by herbicides) where wild soybean occurs [4]. Insect-protected soybean has been developed to protect against leaf feeding by certain Lepidopteran insects [29]. As part of the ERA for Japan a potential hazard was hypothesized as a reduction in leaf feeding by Lepidopteran insects leading to an increased weediness of wild soybean with an introgressed insect-protection trait from soybean.

One indicator of increased weediness and invasiveness may be a higher number of wild soybean individuals in an area [11, 30]. Although the environmental and biological factors that currently limit wild soybean populations are not known, an effective ERA of insect-protected soybean would, include scientific focus on the effects of Lepidopteran leaf feeding insects on wild soybean populations. In order to evaluate this potential effect, the amount of herbivory by Lepidopteran insects under natural conditions, as well as the ability of wild soybean to tolerate insect herbivory, needed to be quantified.

Surveys of insects that feed on wild soybean in Japan have been conducted previously. Wild soybean under natural conditions in Japan and reported that 47 Lepidopteran species fed on wild soybean [31]. However, defoliation of wild soybean is not exclusively performed by Lepidopteran insects, because species of Orthopteran, Hemipteran, Coleopteran, and Dipteran orders also feed on wild soybean [32]. No quantitative information of defoliation caused by these insects has been reported and it is not known if insect feeding and more specifically feeding by Lepidopteran insects, limits the weediness or invasiveness of wild soybean.

In order to assess the potential impact of insect leaf feeding on wild soybean populations in Japan it was important to consider the potential for wild soybean to compensate for defoliation. Many plant species are known to have compensatory responses that can limit the impact of leaf herbivory on characteristics like seed production [33]. Defoliation at vegetative stages [34, 35] or at late reproductive stages [34] generally does not affect seed yield of cultivated soybean. However, early- to mid-reproductive stages are more sensitive to defoliation and may result in reduction of yield or yield-related characteristics [34–36]. Studies have shown that cultivated soybean can compensate for up to 20% leaf loss without a reduction in seed production [35–37]. Wild soybean may have a similar ability to compensate for defoliation by insects, but to our knowledge, no empirical data has been reported.

Two studies were conducted to assess the hypothetical weediness potential of wild soybean in the unlikely event that hybridization with a GM insect-protected soybean would result in GM trait integration: 1) a survey of natural wild soybean populations to determine which insect herbivore species feed on wild soybean and what level of defoliation they cause, and 2) an experiment to assess the impact of defoliation on wild soybean seed and pod production. The results of both of these studies may be used by risk assessors to evaluate a potential hazard (environmental consequence) from the unlikely introgression of an insect-protection trait from cultivated soybean into wild soybean.

## Materials and Methods

### Field survey of wild soybean populations: Assessment of percent defoliation caused by herbivory

Populations of naturally growing wild soybean were identified at Tsukuba-shi, Shimotsuma-shi, and Yachiyo-shi locations in Ibaraki prefecture, and Saga-shi, Kanzaki-shi, and Misaki-machi locations in Saga prefecture. The habitat description for each surveyed population is listed in Table 1. These survey areas were considered representative of environments where natural wild soybean populations occur in Japan [38].

**Table 1:** Habitat for wild soybean populations observed in the surveys of insect leaf feeding damage in 2011, 2012 and 2013 in Japan.

Area (Prefecture)	Population number	Observation year	Description of habitat where wild soybean population was observed
	1	2011, 2012, 2013	Grass field generated with the construction of Tsukuba bypass near or on Route 125
	2–10	2011	
	11	2011	Slope along agricultural water way
	12	2011, 2012, 2013	Dike
	13	2011	Dike
	14	2011, 2012, 2013	Fallow soil
	15	2011	Fallow soil
Ibaraki	16	2011, 2012, 2013	Grass field with gravelly soil including heap from other place
	17	2011	Empty lot next to a field
	18	2011	Dike along agricultural waterway
	19	2011, 2012, 2013	Fallow soil
	20	2011	Fallow soil
	21	2011, 2012, 2013	Fallow soil
	22	2011, 2012, 2013	Empty lot at field side, heap
	23	2011, 2012, 2013	Empty lot at field side
	1	2011, 2012, 2013	Unmanaged slope along a creek
	2	2011	Well managed slope along a creek
	3	2011, 2012, 2013	Managed slope along a creek
	4	2011	Well managed slope along a creek
	5	2011, 2012, 2013	Partially managed slope along a creek
	6	2011, 2012, 2013	Well managed slope along a creek
Saga	7	2011, 2013	Well managed slope along a creek
	8	2011	Well managed slope along a creek
	9	2011	Soil spoiled area
	10	2011, 2012, 2013	Unmanaged slope along a creek
	11–12	2011	Unmanaged slope along a creek
	13	2011	Slope along a creek, construction site
	14–16	2011, 2013	Roadside, under new construction
	17	2011	Well managed slope along a creek

Typically, wild soybean emerges from April to August [39] and matures from October to December [40]. However, the rate of growth and development differs by region and environmental conditions from year to year. The survey was initiated on June 2011, when wild soybean populations were most likely to have emerged and could be found. The observations continued every two weeks until the plants matured. At each observation the growth stage of the wild soybean plants was documented as vegetative (VE—Vn), or reproductive (flowering at R1-R2 and pod/seed development from R3 to R8) [41].

**Fig 1:**
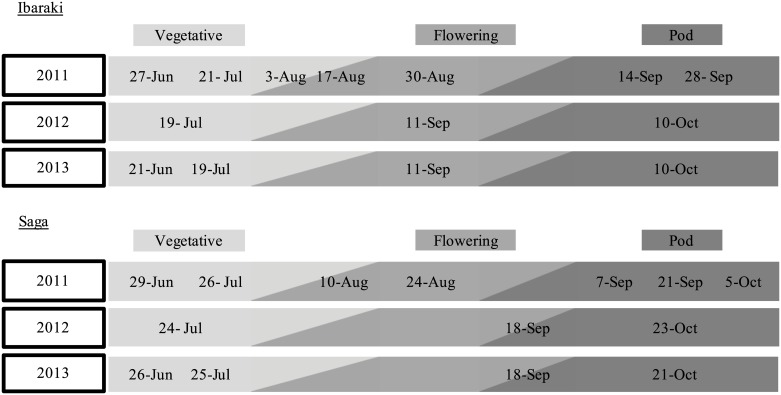
The growth stages of wild soybean at each observation time at each site during each year. Growth stages were defined as; Vegetative (VE—Vn), Flowering (R1 –R2), and Pod/seed development (R3 –R8) as described by Pedersen [41]. The date associated with the bars denotes the observation date at each site. Observation date including two different colors (*e*.*g*., 10-Aug., 2011) indicates that the growth stage of wild soybean varied among plants of the same population at that time.

Based on the observations from the 2011 season, the surveys were optimized for the 2012 and 2013 seasons to ensure at least one observation was conducted during each of the vegetative, flowering, and pod/seed development phases. The dates of observations and growth stages are listed in Fig 1.

From July to November of each year (2011–2013), a visual assessment of the wild soybean plants was made of foliage damage caused by different herbivore groups that were active at each the observation time. A square frame (30cm x 30cm) was randomly placed over wild soybean plants at three different areas within each population at each observation time. Without tissue/plant removal, defoliation and leaf damage was evaluated within the frame using a continuous 0–100% scale of increasing leaf damage severity. The foliage damage symptoms described in Table 2 were used to distinguish feeding damage caused by different herbivore groups.

**Table 2:** Foliar damage attributed to various herbivores based on symptoms and herbivore feeding pattern.

Defoliation			Leaf damage not involving tissue removal
Coleopteran spp.	Orthopteran spp.	Lepidopteran spp.	Hemipteran spp., Homopteran spp., Acari, and Mollusca
Feed on the softer leaf tissue, but usually avoid feeding on even the smallest leaf veins, thereby leaving a leaf “skeleton” (*e*.*g*., Japanese beetles).	Feed on entire leaf tissue except for the tougher leaf veins. Leaves with ragged edges due to irregular cuts and tearing (*e*.*g*., grasshoppers, crickets).	Leaves are often with ragged edges due to more or less smooth cuts and tearing. Prefer to feed on the tops of the plant and usually start their feeding from the border of the leaf blade. Feeding symptoms are also characterized by the presence of feces (most Lepidopteran insects). Leaves are tied together by silken threads (web). The leaves inside the webbings are nearly consumed, except for the tougher leaf veins (*e*.*g*., leaf rollers)	Presence of white spots most noticeable on the upper side of the leaves. The leaves eventually wither and die (*e*.*g*., stalk-eyed bug). Presence of many small yellowish or brownish speckles; cupping and burning of leaf margins (*e*.*g*., leaf hoppers). Presence of leaves puckering or plant stunting; honey dew secretions resulting in the growth of sooty mold (*e*.*g*., aphid). Presence of white or yellow spots most noticeable on the underside of the leaves. The leaves can eventually wither and die (*e*.*g*., spider mites). Presence of slimy, silver-colored trail most noticeable on the underside of the leaves; leaves appear distorted and tattered (*e*.*g*., slugs, snails).

The survey was done in accordance with Japan laws. The surveys and field observations were conducted in ditches and non-protected land, therefore no permits were required. The survey did not involve endangered or protected species.

### Growth Chamber Study: Defoliation effect on wild soybean pods and seed production

The growth chamber study was performed in Saint Louis, Missouri, USA, at Monsanto Company facility. An alternating temperature regime was maintained with 60% humidity at 26°C for 13 hours under light and at 22°C for 11 hours under dark. The experimental design was a randomized complete block with 12 replications.

A total of 150 wild soybean seeds (PI339736A, USDA-GRIN Germplasm Bank), were planted in M360 potting soil media (Hummert International, Earth City, Missouri, USA) in 200-cell trays. When the emerged plants reached the early seedling stage (VC) [41], 60 individuals with uniform growth were selected and transplanted into 14.7 cm diameter pots containing M360 potting soil media. These 60 individuals were randomly assigned into 12 replicates (one plant per replicate) and each plant was randomly assigned to a defoliation treatment of 0%, 10%, 25%, 50%, or 100% of the leaf area. Two plants died prematurely resulting in two treatments (0% and 10%) represented by 11 replicates each. Cultivated soybean is most sensitive to defoliation during the flowering (R1-R2 stage) and pod-fill stages as evidenced by yield reduction [34, 36]. Thus, mechanical defoliation was conducted at the R1-R2 stage (from the beginning of flowering to the full bloom). Defoliation was initiated from the top to the bottom of the canopy over a three day period in order to simulate closely the pattern of Lepidopteran leaf feeding [34, 42]. Each defoliation treatment consisted of removing the appropriate percentage of leaves starting with the upper third of the canopy the first day, followed by leaf removal from the middle of the plant on the second day, and finally leaf removal from the bottom third of the canopy on the third day. The leaves across all treatments were removed by cutting the petiole at the base of each leaflet. The 100% defoliation treatment consisted of removal of all fully expanded leaves on the main stems and branches. The number of pods and seeds for each plant was counted per plant at early maturity (R7).

The experimental part of this study was conducted in compliance with the USA laws and regulations. The defoliation experiment was conducted in confined environment at Monsanto’s facility in St. Louis, MO following state laws. The experimental materials originated from the seed obtained from the USDA-GRIN germplasm bank. The experiment did not involve endangered or protected species.

### Statistical Analysis

#### Field survey of wild soybean populations

Among organisms feeding upon wild soybean, Coleopterans, Orthopterans, and Lepidopterans involved in defoliation were consistently observed across all populations within each prefecture allowing for statistical analysis. An analysis of variance (ANOVA) was performed using SAS^®^ version 9.4[43]. A factor “Environment” was created by concatenating the site with the year of the observations. The observations within an “Environment” were grouped by each of the three growth stages (“Vegetative”, “Flowering”, and “Pod/Seed development”). If a growth stage within a year contained multiple observations, the average was used for the analysis. A log_10_ (1+X) variance stabilizing transformation was utilized to satisfy the ANOVA assumptions. Pairwise comparisons between Coleopterans, Orthopterans, and Lepidopterans within each prefecture, year, and growth stage were defined within the ANOVA and tested using t-tests. The level of statistical significance was predetermined to 5% (α = 0.05).

#### Growth Chamber study

An analysis of variance for the numbers of pods and seeds per plant at the R7 (beginning maturity) growth stage was performed using SAS^®^ version 9.2 [44]. Pairwise comparisons between undefoliated control and 10%, 25%, 50%, or 100% defoliation treatments were defined within the ANOVA and tested using t-tests. The level of statistical significance was predetermined to 5% (α = 0.05).

## Results

### Level of defoliation

Across all populations and growth stages, it was observed that many organisms fed upon wild soybean, causing defoliation and leaf damage (Table 2). The major taxa included species from the: Orthoptera order (*e*.*g*., grasshopper and field cricket), Coleoptera order (*e*.*g*., Japanese beetle and leaf beetles), and Lepidoptera order (*e*.*g*., *Spodoptera litura*, *Matsumuraeses falcana*, loopers). When assessed across all populations within each prefecture, the feeding from these three orders contributed less than 7.75% to foliar damage, with the highest levels of defoliation caused by Orthopterans, followed by Coleopterans (Table 3). Levels of defoliation from Lepidopterans were very low at all growth stages, years, and prefectures (locations) and in most cases lower than defoliation by Orthopterans and Coleopterans (Table 3).

**Table 3:** Comparisons of three major herbivore groups feeding on wild soybean.

Prefecture	Year	Growth Stage	Defoliation (%)		
			Coleopteran spp.	Orthopteran spp.	Lepidopteran spp.
Ibaraki	2011	Vegetative	3.73 b	6.91 a	0.77 c
		Flowering	0.98 b	2.63 a	0.93 b
		Pod/Seed Development	0.40 c	2.30 a	1.27 b
	2012	Vegetative	0.70 a	0.89 a	0.01 b
		Flowering	0.14 b	0.85 a	0.00 b
		Pod/Seed Development	0.03 b	2.75 a	0.04 b
	2013	Vegetative	3.07 a	0.71 b	0.01 c
		Flowering	0.20 a	0.60 a	0.10 a
		Pod/Seed Development	3.37 a	3.22 a	0.07 b
Saga	2011	Vegetative	3.73 b	7.75 a	1.66 c
		Flowering	0.55 b	1.68 a	0.80 b
		Pod/Seed Development	0.17 c	1.51 a	0.79 b
	2012	Vegetative	1.46 a	0.81 a	0.08 b
		Flowering	0.07 a	0.20 a	0.09 a
		Pod/Seed Development	0.18 b	1.97 a	0.09 b
	2013	Vegetative	2.95 a	1.25 b	0.24 c
		Flowering	0.36 ab	1.46 a	0.13 b
		Pod/Seed Development	1.07 ab	1.41 a	0.58 b

Note: Values in each row followed by a different letter group indicated significant difference (p<0.05).

**Fig 2:**
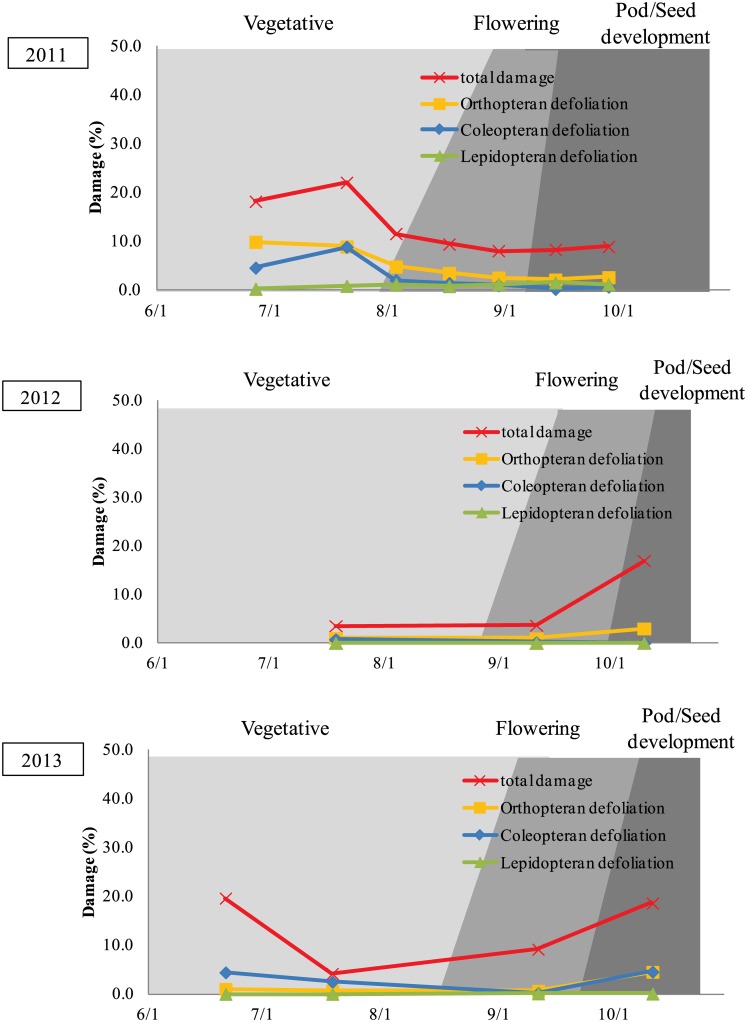
Observed level of defoliation by different insect taxa and the total insect damage by all organisms at Ibaraki in 2011, 2012, and 2013.

**Fig 3:**
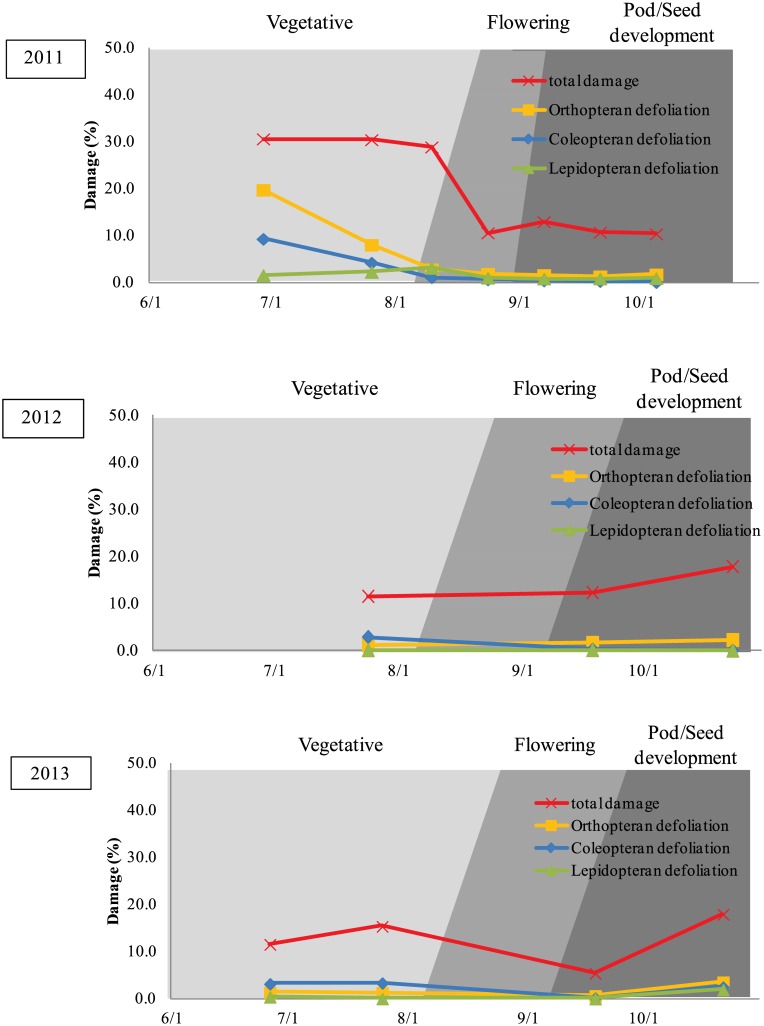
Observed level of defoliation by different insect taxa and the total insect damage by all organisms at Saga in in 2011, 2012 and 2013.

Herbivore injury to wild soybean varied depending on the environmental (site and year). The total foliage damage across sites and herbivore group ranged from 3.6–30.6% (Figs 2 and 3). In 2011, foliar damage was the highest during the early part of the growing season (June and July) in both Ibaraki and Saga, but in 2012 and 2013, the levels of foliar damage remained unchanged or increased later in the growing season (September/October).

At Ibaraki prefecture in 2011, total foliar damage, reflecting defoliation by all herbivore groups plus other leaf damage not involving tissue removal, was the highest (22.1%) at the second observation during the vegetative stage and the lowest (8.0%) at the fifth observation during the flowering stage (Fig 2). The percent defoliation from Orthopterans was the highest (9.9%) at the first observation during the vegetative stage and the lowest (2.1%) at the sixth observation during the pod/seed development stage (Fig 2). The percent defoliation from Coleopterans was the highest (8.8%) at the second observation during the vegetative stage and the lowest (0.2%) at the sixth observation during the flowering stage (Fig 2). Compared to Orthopterans, the percentage of defoliation by Lepidopterans was lower at all observations throughout the growing season. Compared to Coleopterans, the percentage of defoliation by Lepidopterans was lower at the first four observations during the vegetative and flowering stages and was slightly higher at the fifth to seventh observations during the pod/seed development stage, but never exceeded 2% defoliation (Fig 2).

At Ibaraki prefecture in 2012, total foliar damage of wild soybean was the highest (17.0%) at the third observation during the pod/seed development stage and the lowest (3.6%) at the first observation during the vegetative stage. The percent defoliation from Orthopterans was the highest (3.0%) at the third observation during the pod/seed development stage, and was the lowest (1.0%) at the second observation during the flowering stage (Fig 2). The percent defoliation from Coleopterans was the highest (0.8%) at the first observation during the vegetative stage and was the lowest (<0.1%) at the third observation during the pod/seed development stage (Fig 2). Compared to Orthopterans or Coleopterans, the percent defoliation from Lepidopterans was consistently low (< 0.1%) at all observations throughout the growing season (Fig 2).

At Ibaraki prefecture in 2013, total foliage damage was the highest (19.6%) at the first observation during the vegetative stage and the lowest (4.2%) at the second observation during the vegetative stage. The defoliation from Orthopterans was the highest (4.5%) at the fourth observation during the pod/seed development stage, and was the lowest (0.7%) at the third observation during the flowering stage. The defoliation from Coleopterans was the highest (4.6%) at the fourth observation during the pod/seed development stage and was the lowest (0.2%) at the third observation during the flowering stage. Compared to Orthopterans or Coleopterans, percent defoliation from Lepidopterans was consistently low (< 0.1%) at all observations throughout the growing season (Fig 2).

At Saga prefecture in 2011, total foliar damage was the highest (30.6%) at the first observation during the vegetative stage and the lowest (10.3%) at the seventh observation during the pod/seed development stage. The percent defoliation from Orthopterans was the highest (19.7%) at the first observation during the vegetative stage and the lowest (1.3%) at the sixth observation during the pod/seed development stage. The percent defoliation from Coleopterans was the highest (9.3%) at the first observation during the vegetative stage, and the lowest (below 0.1%) at the seventh observation during the pod/seed development stage. The percent defoliation from Lepidopteran was the highest (3.1%) at the third observation during the vegetative/flowering stage and lowest (0.8%) at the fifth observation during the pod/seed development stage. Compared to Orthopterans, the percent defoliation from Lepidopterans was lower at all observations throughout the growing season. Compared to Coleopterans, the percent defoliation from Lepidopterans was lower for the first two observations during vegetative stage and was slightly higher for third to seventh observations during the vegetative, flowering, and pod/seed development stages, but never exceeded 2% defoliation (Fig 3).

At Saga prefecture in 2012, total foliage damage was the highest (17.9%) at the third observation during the pod/seed development stage and the lowest (11.6%) at the first observation during the vegetative stage. The percent defoliation from Orthopterans was the highest (2.3%) at the third observation during the pod/seed development stage and was the lowest (1.2%) at the first observation during the vegetative stage. The percent defoliation from Coleopterans was the highest (2.9%) at the first observation during the vegetative stage and was the lowest (< 0.1%) at the third observation during the pod/seed development stage. Compared to Orthopterans or Coleopterans, the percent defoliation from Lepidopterans was consistently low (< 0.1%) at all observations throughout the growing season (Fig 3).

At Saga prefecture in 2013, total foliage damage was the highest (18.0%) at the fourth observation during the pod/seed development stage and the lowest (5.4%) at the third observation during the flowering and pod/seed development stages. The percent defoliation from Orthopterans was the highest (3.6%) at the fourth observation during the pod/seed development stage and was the lowest (0.7%) at the third observation during the flowering and pod/seed development stages. The percent defoliation by Coleopterans (3.4%) was the highest at the second observation during the vegetative stage and was the lowest (0.2%) at the third observation during the flowering and pod/seed development stages. The percent defoliation from Lepidopterans was the highest (1.9%) at the fourth observation and was the lowest (< 0.1%) at the third observation during the pod/seed development stage. Compared to Orthopterans or Coleopterans, the percent defoliation from Lepidopterans was consistently lower at all observations throughout the growing season (Fig 3).

### Defoliation effect on number of pods and seeds

**Fig 4:**
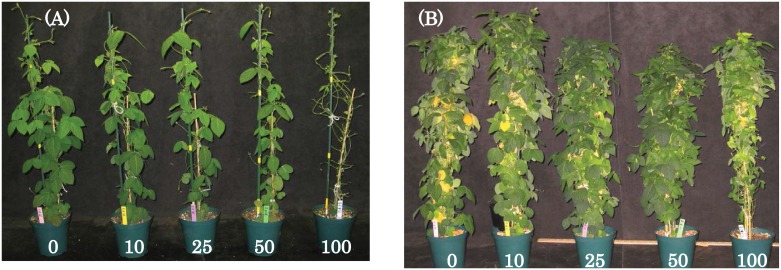
Example of wild soybean plants at R1-R2 growth stage just prior to defoliation treatments (A) and after defoliaton treatments at the R7 growth stage (B). The number in the figure denotes the percentage of mechanical defoliation. No defoliation (0%) treatment was used as the control.

Wild soybean plants grew as expected both prior to and after transplanting and the appearance of the plants before and after the defoliation treatments is shown in Fig 4. There were no observed differences in morphology among the 60 plants randomly selected at the early vegetative stage (VC growth stage) to early flowering (R1-R2 growth stage) when the defoliation treatments were applied.

**Fig 5:**
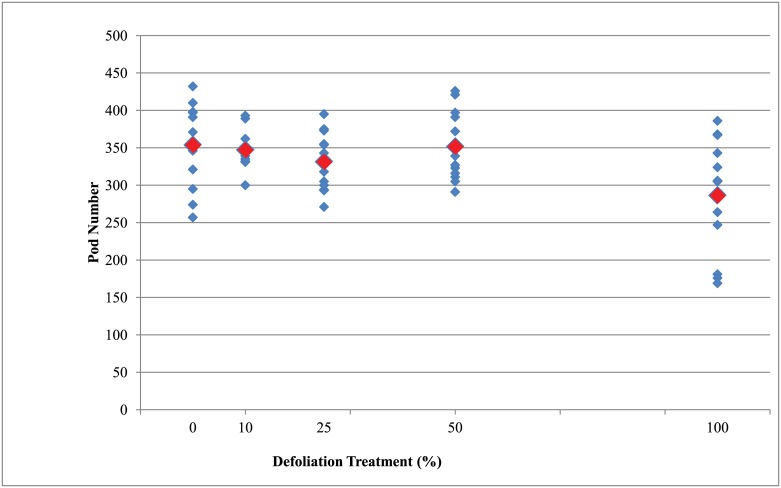
The number of pods produced per individual wild soybean plant after different defoliation treatments. The blue diamonds are the total number of pods per plant and the red diamonds are the mean number of pods across all plants within each defoliation treatment.

**Fig 6:**
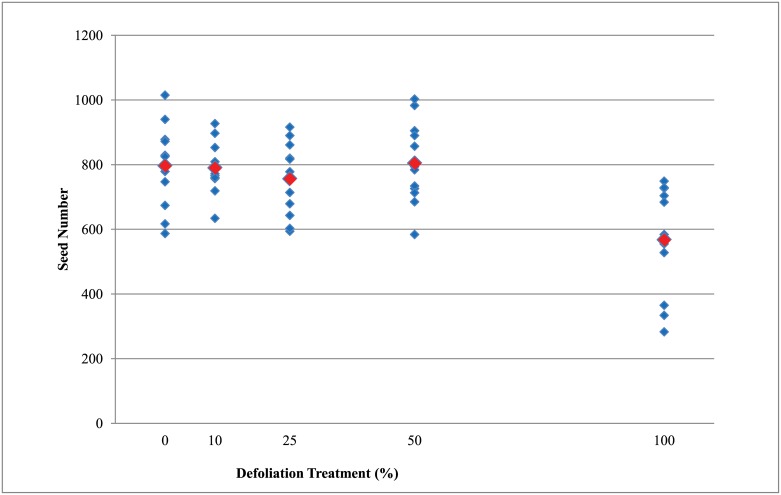
The number of seed produced per individual wild soybean plant after different defoliation treatments. The blue diamonds are the total number of seeds per plant and the red diamonds are the mean number of seeds across all plants within each defoliation treatment.

The numbers of pods and seeds produced by plants in the 100% defoliation treatment were significantly lower (p<0.05) than the undefoliated control (0% defoliation) plants (Table 4, Figs 5 and 6). The number of pods and seeds produced by plants with 10%, 25%, and 50% defoliation were not significantly different from the undefoliated control plants. The number of days to reach early maturity (R7 growth stage) was numerically greater for the plants with 100% defoliation relative to the undefoliated control. In contrast, plants with 10%, 25%, and 50% defoliation did not differ in the number of days to reach early maturity (R7) relative to the undefoliated control (Table 4).

**Table 4:** The number of pods and seeds per plant, days to flowering and days to early maturity of wild soybean after defoliation treatments.

Defoliation treatment (%)	Number of pods / plant		Number of seeds / plant		Days to flowering^3^	Days to early maturity^4^
	Mean (SE)^1^	Range^2^	Mean (SE)^1^	Range^2^		
0 (undefoliated control)	354 (17.8)	257–432	797 (40)	587–1,015	30	68
10	347 (7.9)	300–393	790 (25)	634–927	30	68
25	331 (11.4)	271–395	756 (32)	594–916	30	68
50	352 (13.6)	291–426	805 (36)	584–1,003	30	68
100	286 (22.6)*	169–386	568 (47)*	283–749	30	78

* Indicates a significant difference between each defoliation treatment relative to the undefoliated control (p<0.05).

^1^SE = standard error

^2^Minimum and maximum values observed within each defoliation treatment.

^3^Days to flowering (R1 –R2 growth stage) when the defoliation treatments were applied. R1 = Open flower at any node on the main stem, and R2 = Open flower at one of the two uppermost nodes on the main stem with a fully developed leaf.

^4^Days to early maturity (R7 growth stage) when harvest was conducted. R7 = One normal pod on the main stem that has reached its mature pod color.

## Discussion

There have been 10 herbicide-tolerant GM soybean traits approved for importation to Japan [4]. The Ministry of Agriculture, Forestry and Fisheries and Ministry of Environment in Japan determined from the ERAs of these GM soybeans that if an herbicide-tolerance trait were transferred to wild soybean it would likely have a neutral effect on these populations because they are typically found in areas where herbicides are not applied. Thus, the potential for increased weediness or invasiveness of wild soybean from herbicide-tolerance traits is negligible [4]. However, it was not know how an insect-protection trait might influence wild soybean populations in Japan.

Insect-protected soybean has been developed to protect against leaf feeding by some Lepidopteran insects. Wild soybean is a cross-compatible relative of soybean that grows naturally in Japan. The assessment of potential increased weediness or invasiveness of wild soybean (hazard) in the unlikely even that an insect-protected transgene was introgressed from soybean to wild soybean was one aspect considered in the environmental risk assessment for Japan.

Similar to other plants species, expansion of wild soybean populations is limited by numerous factors including plant-to-plant competition, interaction with abiotic factors in the environment (*e*.*g*., drought, nutrient stress), interactions with other organisms (*e*.*g*., herbivores, diseases) and human activity [45]. In this study we quantitatively evaluated the levels of foliar damage of wild soybean in naturally occurring populations in Japan. Total foliar damage from all herbivores (leaf damage not involving tissue removal) of wild soybean populations throughout the growing seasons at all sites and years was 30% or less. Among the observed herbivores, defoliation from Lepidopteran insects was very low (averaging less than 2% across locations and years) compared to defoliation caused by other herbivores. The results of these observations, which indicate that numerous types of herbivores (from the orders of Orthoptera, Coleoptera, Lepidoptera, Hemiptera, Homoptera, and Diptera) feed on wild soybean leaves, which is in agreement with previous studies conducted in Japan [31, 32]. The limited defoliation by Lepidopterans observed at Ibaraki and Saga relative to defoliation caused by other taxa, would suggest that Lepidopterans alone are not the primary herbivores feeding on wild soybean leaves in Japan.

The result from the simulated herbivory feeding study demonstrated that greater than 50% defoliation at flowering (R1-R2 growth stage) may significantly reduce pod and seed production and delay maturity of wild soybean. The plants compensated for up to 50% defoliation by producing new leaves and were able to produce equivalent numbers of pods and seeds as the undefoliated control plants. However, the complete removal of all leaves (100% defoliation) at flowering (R1-R2 growth stage) resulted in a significant decrease in the number of pods and seeds and delayed maturity by more than 10 days relative to undefoliated plants. These results are in agreement with Fehr, Caviness [46], who reported that 100% defoliation of cultivated soybeans at the R2 growth stage resulted in 13% to 39% reduction in seeds produced per plant. Delayed senescence has been observed in previous defoliation studies conducted with cultivated soybeans [34, 47]. Similar trends were observed for both pod and seed production for wild soybean response to defoliation. Similarly, in soybean it has been shown that pod number is highly correlated with seed number [47, 48]. However, the tolerance of soybean to defoliation seems to be lower than that of wild soybean during reproductive stages. In this study, wild soybeans tolerated up to 50% defoliation without an impact on pod or seed number, whereas tolerance by cultivated soybean to defoliation without economic injury is approximately 15–20% [35–37] during the reproductive developmental stages. Shapiro, Peterson [49] estimated the percent yield loss of indeterminant soybean varieties by different levels of defoliation at R1-R2 growth stage. Their data suggests that soybeans can tolerate up to 10% defoliation without yield losses, but will suffer 2%, 3% or 6% yield reduction with 20%, 30% or 50% defoliation, respectively. Our results indicate that wild soybean is more tolerant to defoliation than cultivated soybeans. Welter and Steggall [50] speculated that domestication of tomatoes decreased their tolerance to herbivory compared to their wild relative. Our results support the hypothesis that similar processes might have happened during soybean domestication, resulting in soybean’s reduced tolerance to herbivory as compared to wild soybean.

Plant compensatory responses to defoliation involves mechanisms related to allocation and reallocation of resources [33], with some response mechanisms to defoliation including changes in re-growth pattern, photosynthetic activity, delay of leaf senescence, changes in leaf shape, and canopy architecture [33, 42]. Overall, the results from the defoliation study show that wild soybean exhibits a high tolerance to defoliation. No significant reduction in wild soybean pod or seed number or time to reach maturity were observed up to 50% defoliaton compared to the undefoliated control. The defoliation tolerance of wild soybean was achieved mainly because plants tended to compensate for defoliation by producing new leaves (Fig 4), and/or by delaying senescence (Table 4). This compensation strategy is similar to that observed in studies with cultivated soybean [34, 42, 51].

It has been reported that indeterminate type soybean cultivars have a higher compensatory responses to defoliation than determinate cultivars by producing more leaves even during the reproductive stage [42]. Wild soybeans are indeterminate and grow and develop over a longer periods of time during the season than soybeans. Thus, wild soybean has the potential to branch more and produce more leaves during both vegetative and reproductive stages which may buffer against leaf losses and thus maintain a compensatory ability to produce seed.

The total insect damage observed at each site varied among the three years of the survey. There are several possible reasons for this variation. One explanation might be that environmental conditions (*e*.*g*., nutrients and water availability) were better for plant growth in some years than others, which can improve the ability of plants to compensate for insect defoliation [33]. For example, at Ibaraki and Saga in 2011, it was observed that herbivore level decreased as the growth stage advanced. Assuming that the wild soybean plants were exposed to the comparable number and level of insect herbivory from vegetative to flowering stages, it may be that the rate of producing new leaves surpassed the losses by herbivory and, consequently, the observed herbivory level appeared to be lower later in the season. Although wild soybean is found mainly near river basins or downriver of paddy fields [19–24] where water supply is sufficient, excess rainfall would negatively impact growth of wild soybean. At Ibaraki and Saga in 2011, the rainfall in May was above the average rainfall for recent 10 years. In contrast, the rainfall in July and August was similar to the average rainfall for recent 10 years [52]. The rainfall in May, early vegetative stage for wild soybean may negatively impact to growth and, appearance of the total insect damage may be larger than other observation years. The adequate rainfall in late vegetative and early reproductive stage advance production of new leaves.

Additional explanations for describing the observed variation are 1) synergy and antagonism of particular arthropods and pathogens on wild soybean response to defoliation, which has been extensively reviewed by Hauser, Christensen [53], 2). wild soybean defoliation response that did not differ from year to year, but insect dispersion pattern or timing of insect lifecycle may have differed from season to season [33]. Although wild soybean growth stages occurred at similar calendar dates across years, there was variation that may account for different movement and feeding by insects from year to year. There may be other plausible explanations for the observed variation in wild soybean defoliation observed among years and sites in this study.

It is reported an increase in seedling density per unit area of wild soybean did not affect the seeds produced per unit area [54]. Even if seed production was decreased due to defoliation by herbivory, it would not likely affect the population size in the next year because wild soybean produces excess seed, and their population size seems to be influenced more by environmental factors (*i*.*e*., appropriate microsites for promoting germination) and self-thinning than by seed output.

The potential to increase the weediness or invasiveness of wild soybean is low for an unlikely introgressed Lepidopteran-protection trait because defoliation by Lepidopterans is: 1) very low and 2) below levels that would reduce pod and seed production. It is unlikely that Lepidopteran insects are limiting the distribution and abundance of wild soybean populations in Japan. Therefore, the potential environmental consequence of a Lepidopteran-protection trait introgressed into wild soybean from imported soybean is limited.

## Conclusions

The studies presented in this report indicate that Lepidopteran leaf feeding on wild soybean in Japan is very low (< 2%) and that total foliage damage by all herbivores on wild soybeans is low (< 30%). Furthermore, wild soybeans have an ability to compensate for substantial defoliation. This study has demonstated that up to 50% defoliation at flowering resulted in no delay in maturity, and no reduction in seed and pod production. Thus, in the unlikely event that an insect-protection trait were introgressed into wild soybean in Japan, it would not lead to increases in weediness or invasiveness of wild soybean. Thus, the potential consequence component of the ERA of an insect-protected soybean imported to Japan is considered to be extremely low.
